# Comparing the effectiveness of copper intrauterine devices available in Canada. Is FlexiT non-inferior to NovaT when inserted immediately after first-trimester abortion? Study protocol for a randomized controlled trial

**DOI:** 10.1186/1745-6215-13-147

**Published:** 2012-08-24

**Authors:** Wendy V Norman, Jessica L Chiles, Caroline A Turner, Rollin Brant, Andra Aslan, Janusz Kaczorowski

**Affiliations:** 1 Contraception Access Research Team, Women’s Health Research Institute, Vancouver, British Columbia, V6H 1G3, Canada; 2 Department of Family Practice, University of British Columbia, Vancouver, British Columbia, V6T 1Z3, Canada; 3 School of Population and Public Health, University of British Columbia, Vancouver, British Columbia, V6T 1Z3, Canada; 4 Department of Statistics, University of British Columbia, Vancouver, British Columbia, V6H 3V4, Canada; 5 Département de médecine familiale et médecine d’urgence, L’Université de Montréal, Montréal, Quebec, Canada

**Keywords:** Contraception, Intrauterine device, Intrauterine device expulsion, Contraception effectiveness, Abortion-induced, Therapeutic abortion, Non-inferiority trial, Randomized controlled trial, Canada

## Abstract

**Background:**

We describe the rationale and protocol for a randomized noninferiority controlled trial (RCT) to determine if the Flexi-T380(+) copper intrauterine contraceptive device (IUD) is comparable in terms of effectiveness and expulsion rates to the most common Canadian IUD currently in use, NovaT-200, when placed immediately after a first-trimester abortion.

**Methods/Design:**

Consenting women choosing to use an IUD after an abortion for a pregnancy of less than 12 weeks of gestation will be randomized to device-type groups to receive immediate post-abortion placement of either a Flexi-T380(+) IUD, a device for which no current evidence on expulsion or effectiveness rates is available, or the Nova-T200 IUD, the only other brand of copper IUD available in Canada at the time of study initiation. The primary outcome measure is IUD expulsion rate at 1 year. Secondary outcomes include: pregnancy rate, method continuation rate, complication rates (infection, perforation), and satisfaction with contraceptive method. A non-intervention group of consenting women choosing a range of other post-abortion contraception methods, including no contraception, will be included for comparison of secondary outcomes. Web-based contraception satisfaction questionnaires, clinical records, and government-linked health administrative databases will be used to assess primary and secondary outcomes.

**Discussion:**

The RCT design, combined with access to clinical records at all provincial abortion clinics, and to information in provincial single-payer linked administrative health databases, birth registry, and hospital records, offers a unique opportunity to determine if a novel IUD has a comparable expulsion rate to that of the current standard IUD in Canada, in addition to the first opportunity to determine pregnancy rate and method satisfaction at 1 year post-abortion for women choosing a range of post-abortion contraceptive options. We highlight considerations of design, implementation, and evaluation of the first trial to provide rigorous evidence for the effectiveness of current Canadian IUDs when inserted after first-trimester abortion.

**Trial registration:**

ClinicalTrials.gov Identifier NCT01174225

## Background

This randomized non-inferiority controlled trial (RCT) will examine the effectiveness of post-abortion contraception by determining whether a new type of copper intrauterine device (IUD) in Canada has a comparable effectiveness and safety profile to the most commonly used Canadian IUD. Clinical experience suggests that expulsion rates may vary significantly between devices, but we have been unable to find any published evidence estimating expulsion rate or pregnancy rate for this new IUD. Our results will provide some evidence of whether the new IUD is comparable with the current standard, and provide the first prospective rigorous evaluation of effectiveness at 1 year for post-abortion contraceptive methods currently available in Canada.

Nearly a third of Canadian women have at least one abortion [1]. Annually about 100,000 abortions are performed in Canada [2]. In 2009, at least 30.7% of these were repeat abortions [3]. Women having repeat abortions are more likely to be from ethno-cultural minorities, to report problems with a male partner, to have a lower level of education, and to have other children at home [4,5]. Thus, research to delineate methods to prevent recurrent unwanted pregnancies in this vulnerable population is a high priority. Ames [6] found a 5-year repeat abortion rate of 9.4% for Canadian women who had an IUD (Flexi-T380(+)®; Prosan International BV. Arnhem, The Netherlands) [7] placed immediately after a first-trimester abortion. However, this retrospective study was only able to determine the recurrent abortion rate of women returning to the index abortion clinic; thus, it is possible that the number of pregnancies was significantly higher than the number of repeat abortions reported. Even so, the reported repeat abortion rate of 9.4% is significantly higher than would be expected based on evidence for the post-abortion effectiveness of a copper IUD [8,9]. Overall 5-year pregnancy rate for IUD users is estimated to be less than 2% [10-12], or a repeat abortion rate of 35 per 1000 women-years of follow-up for women with other models of IUD placed post-abortion [13]. Indeed, the placement of copper IUD types available in other jurisdictions as an effective contraception method following a first-trimester (before 12 weeks of gestational age) abortion is well supported by systematic reviews [8,14].

The only copper IUDs available in Canada at the time this study began were the Nova-T (Nova-T200®; Bayer Inc, Canada), and two Flexi-T devices (Flexi-T300® and Flexi-T380(+)®; both Prosan International BV) [7]. Canadian physicians report higher rates of expulsion with the Flexi-T devices [6,15] and yet many prefer the Flexi-T380(+) because evidence supports copper devices with a surface area of 380 mm as being more effective than those with an area of 200 mm [10,14,16-19]. We found no reports in the literature describing the expulsion rates or effectiveness for the Flexi-T devices, although reported outcomes for NovaT200 are available [16-19].

This RCT will determine if the Flexi-T380(+) copper IUD (referred to hereafter as ‘FlexiT’) is comparable with the most common Canadian copper IUD currently in use, the NovaT-200 copper IUD (referred to as the ‘NovaT’), when placed immediately after a first-trimester abortion. Additionally, we will report the first rigorous evidence for the effectiveness of, and satisfaction with, the range of post-abortion contraceptive options available to Canadian women.

## Methods/Design

### Study design

This prospective non-inferiority randomized controlled study will recruit women at the time of an abortion at gestational age of less than 12 weeks (first trimester). Consenting women choosing to use an IUD post-abortion will be randomized to type of device, and women choosing other post-abortion contraceptive methods, or no method of contraception, will be enrolled in a non-intervention, observational arm. Primary outcome will be rate of expulsion at 1 year. Secondary outcomes will include rates for pregnancy; complications such as infection or perforation; continuation of, and satisfaction with, the chosen contraceptive method as determined at 1, 2, 3, 4 and 5 years; and expulsion rate for each type of copper IUD at 2, 3, 4 and 5 years.

### Sample-size justification for comparing expulsion rates for the NovaT and FlexiT

Our aim in this non-inferiority study is to provide evidence that the expulsion rate for the Flexi-T does not exceed that of the Nova-T by more than 10%. The rationale for this choice rests on the assumption that the detection rate for IUD expulsions is at least 85% [20], which implies that the loss in efficacy of a device due to expulsions is less than 0.15 times its expulsion rate, and correspondingly, that any difference in efficacy between devices is less than 0.15 times the difference in expulsion rates. Thus differences of 10% in expulsion rates translate into differences in efficacy of 1.5% or less.

We calculated power calculations for non-inferiority testing following the approach described by Blackwelder [21] using the on-line calculator provided by S. Patten [22].

Our null hypothesis is: H_0_: FlexiT expulsion rate ≥ NovaT expulsion rate + 10%.

This formula requires specification of the problem in terms of success proportions (that is, those not experiencing expulsion by 1 year). We will assume the rate of those not experiencing expulsion will be 96% for the Nova-T [17-19] and 92% for the Flexi-T. Thus, we require a sample size of 192 per group to attain 80% power at α = 0.05 to detect a difference in expulsion rates exceeding 10%. We will enroll a total of 400 women, a sufficient number to allow for changes to intention to leave the province and thus loss to follow-up using the health administrative databases. This recruitment will ensure the final sample is at least the 384 women required to test the null hypothesis that FlexiT has a significantly inferior (higher) expulsion rate compared with the NovaT.

Thus, our recruitment will include 400 women who choose to have IUDs and consent to be randomized to either the Flexi-T (200 women) or the Nova-T (200 women), and a further 200 who decide to not to use intrauterine contraception, who will be enrolled as a non-intervention comparator group. Therefore, a total of 600 women will be recruited.

The study clinic (see ‘Recruitment facility’, below) provides abortions to about 700 women meeting the entry criteria each year, and based on our previous research in this setting [23] we expect 40% of eligible women to choose an IUD, and 80% of these to enroll in IUD groups. Target enrollment is anticipated at 22 months.

### Inclusion criteria

All women at participating study sites who have completed informed consent for an abortion at less than 12 weeks gestational age (as determined by ultrasonography) and who are residents of the Canadian province of British Columbia (BC) enrolled in the universal provincial medical services plan are eligible to participate. Additionally, those who have chosen to use an IUD for post-abortion contraception are eligible to be randomized to the device groups. The study has no minimum age criteria for enrollment.

### Exclusion criteria

Women are not eligible if they intend to move from BC within the next year, if they intend to conceive within 1 year, or if they are currently enrolled in another clinical trial. The contraindications to the use of an IUD are also exclusion criteria.

•Uterine cavity anomalies causing distortion of the endometrial canal including fibroids of more than 50 mm, excluding repaired uterine septum.

• Wilson’s disease.

• Current untreated pelvic inflammatory disease, cervicitis, or lower genital tract infection.

• Undiagnosed abnormal uterine bleeding.

• Known uterine or cervical malignancy or cervical dysplasia.

• Bacterial endocarditis.

• Established immunodeficiency (HIV positivity is not an exclusion unless immunodeficiency is present).

• Acute malignancies affecting blood or leukemias.

• Recent trophoblastic disease with raised levels of human chorionic gonatotropin.

Post-randomization exclusion factors for women in the randomized groups include perforation or excessive bleeding at the time of their abortion, failure to undergo the abortion procedure for any reason, or a uterine anomaly detected at the time of the abortion procedure. These exclusions are designed to be only those that, in real-life clinical practice, would preclude a woman from being able to use an IUD as an immediate method of contraception.

### Recruitment facility

Recruitment for this study will be undertaken at a teaching-hospital outpatient abortion service in the Canadian province of British Columbia (BC).

### Enrollment process

All women presenting at the study clinic for an abortion of a pregnancy before 12 weeks gestational age will receive information via the research study web page [24] at the time they book their appointment, and a study information brochure upon check-in. All women will undergo a post-abortion contraception information session with a trained clinic nurse who is not part of the research staff, and the women will decide upon their contraceptive method of choice before they are asked if they wish to participate in the study. Interested potential participants will then be referred for an information session with a trained women’s health research assistant (RA). The RA will explain all study procedures, answer all questions, and complete the informed consent process with women before the time of their abortion, including establishing preferred personal contact methods to ensure follow-up. Enrolled women will complete an intake version of the contraception satisfaction questionnaire (CSQ) on site. We have previously described the development and use of the CSQ [23]. Briefly, this is a detailed instrument eliciting the effectiveness of, degree of satisfaction with, and adverse effects experienced for the range of contraceptive methods available in Canada. The CSQ has been adapted from a published instrument [25,26] for use in post-abortion studies. It has been translated into the three most common non-English languages in our study population, and piloted for accuracy and cultural sensitivity. This trial has been registered at ClinicalTrials.Gov (Identifier: NCT01174225).

Participants choosing a copper IUD will be randomized to receive either the Flexi-T or the Nova-T. Stratified (that is, separate) randomization lists in sequential blocks of sizes of eight will be generated for parous and nulliparous women. Allocation will be performed remotely using a Canadian on-line randomization service (http://www.randomize.net [27]), to maintain strict concealment. Staff will be kept unaware of the randomization design and of stratification factors involved, to mitigate the potential for unblinding of individual blocks. For women choosing an IUD, the study device determined by the randomization will be inserted by their surgeon immediately after the abortion and before the worman leaves the procedure or operating room. Blinding of the surgeon or the woman receiving the IUD is not possible, as the IUDs are distinct in appearance. Even once *in situ*, these two devices have a differing number and consistency of strings palpable at the cervical os. Following standard clinic protocol, all women will undergo PCR testing for chlamydia and gonorrhea before their abortion, and receive 2 g metronidazole as a single observed dose as prophylaxis against postoperative infection [28]. Women deemed to be at higher risk of a sexually transmitted infection (STI) [28] and those with positive PCR results will receive 1 g of azithromycin as well. A post-placement ultrasound image of the IUD *in situ* will be recorded.

Participants choosing no contraception and all other methods of contraception will receive normal instructions from the facility physician for implementation of their contraceptive method, undergo the same STI testing and prophylaxis protocol, and complete an intake CSQ before leaving the abortion facility.

Enrolled participants will complete the subsequent CSQ either by mail or as a web-based questionnaire at 3, 6, and 12 months post-abortion. User-friendly internet-based and paper-based formats are available for each of the four most common languages spoken in our study population.

All recruited women will receive reimbursement for their time in the form of a gift certificate at enrollment and for each completed CSQ submitted. In addition, women choosing to be randomized to an IUD arm will receive their device free of charge.

### Outcome determination

Primary outcome determination will be through data obtained from the provincial government health administrative databases and from the CSQ to determine the expulsion rate for each group at 1 year. Permission will be obtained from participants to use personal health numbers (the unique identifiers used in these databases) and date of birth to search these databases, detailing all subsequent care received in BC within the subsequent year (including IUD removals and re-insertions, and prescriptions for alternate forms of birth control; information on subsequent pregnancies such as miscarriages, abortions, or deliveries; and any hospital re-admissions, surgery or prescriptions dispensed for antibiotics, which may indicate possible complications). The CSQ will collect data on expulsion of IUD, effectiveness of and satisfaction with the contraceptive method and insertion timing assigned, any removal of the IUD, any change to contraceptive method or intention to conceive, any interval pregnancies and their outcomes, and any adverse events. Using both the databases and the CSQ methods for outcome determination over a period of 5 years (the device life for the IUDs) we will determine, with a close to perfect follow-up rate, the intention to treat (ITT) effectiveness of post-abortion contraception in Canada.

### Ethical considerations

This study has received institutional review board approval from the University of British Columbia-Children’s and Women’s Research Ethics Board (H10-00798) and the Interior Health Authority Research Ethics Board (2010-034).

#### Analysis

Primary outcome will be examined as expulsion rate at 1 year for women randomized to receive the FlexiT device compared with women randomized to a NovaT. Secondary outcomes will be examined annually over the 5-year period using an ITT framework for both randomized and non-intervention groups, and will include: pregnancy rate, rate for continuation of method, adverse events (such as infection or perforation), outcomes of those participants who were chlamydia-positive at the time of abortion, and satisfaction with contraceptive method chosen and expulsion rate for years 2 to 5.

### Analysis methods

Because our aim is to examine expulsion rates and establish the non-inferiority (within a margin of 10%) of the Flexi-T compared with the NovaT, the primary analysis will be based on an upper (one-sided) 95% confidence limit for the difference in expulsion rates (FlexiT rate minus NovaT rate). Non-inferiority will be inferred if the confidence limit value is less than 10%. This simple approach is valid so long as no systematic difference in follow-up occurs between groups. As a check, we will also conduct analysis to account for partial follow-up. Because our outcome definition is essentially composite and the relevant risk periods differ by components, Kaplan-Meier estimates for each component event will be determined, and composite estimates will be obtained by summing the estimated cumulative event rates (calculated as 1 minus the survival function) at the time points indicated in our operational definitions. Confidence intervals around the difference in these estimates will be calculated using the bootstrap method.

Rates for all secondary outcomes will be calculated in an ITT framework for events occurring within 1year and within successive years following the abortion, and for pregnancy events, those associated with a conception within a year or successive years. Outcomes will be determined using the CSQ, direct access to clinical follow-up visit records, and billing and procedure coding data from the administrative health-system databases. Multivariate logistic regression will be used to examine demographic, socioeconomic, and obstetric factors in relationship to primary and secondary objectives.

### Survey analysis

The quantitative data from the CSQ will be analyzed using descriptive and correlational statistics. The CSQ contains several scales, providing composite scores that can be used to indicate differences in the secondary outcomes. Open ended questions will be analyzed through content analysis, focusing on key topics.

## Discussion

### Anticipated limitations

#### Changes in intention to conceive

Owing to our exclusion criteria, we will recruit only women who do not intend to conceive within the first year after enrollment. Nevertheless, in this study population with an anticipated mean age of 24 years, we fully anticipate that some women will change their intent to conceive over the first and subsequent study years. We will account for this in two ways. First we ask at each CSQ interview (3, 6, and 12 months) about the participant’s intent to conceive, and second, we will assume that randomization will distribute those who have intended pregnancies within the study period evenly to both arms of the study.

#### Expulsions

Most women will be aware when an IUD expulsion occurs [20], and should it occur, will make arrangements for alternative contraception. This alternative contraception may be a replacement device or a change in contraceptive method. Each participant will be provided with a toll-free number enabling them to contact the principal investigator (WVN) at any time. In addition, the study team will monitor follow-up visits, CSQs, government medical plan billings, and prescription records of alternative contraception prescribed or an IUD removed or inserted. In this manner, we believe we will be able to estimate device expulsion rate with a fair degree of accuracy. To reflect usual contraceptive conditions most accurately in the event of an expulsion, we are not providing a free replacement device. We have stratified at randomization for parity, as this may be a confounding factor in expulsion.

#### Contribution

This is the first study to determine the expulsion rate for the FlexiT380(+) IUD, and to determine if it is non-inferior to the standard IUD available in Canada, the Nova-T 200, for post-abortion contraception. In addition, this study will provide the first ITT evidence on outcomes for a wide range of contraceptive methods currently available in Canada, including consumer satisfaction, method continuation, and pregnancy rates at 1 year post-abortion. Understanding of satisfaction and effectiveness for common contraceptive options will better inform efforts to prevent recurrent unintended pregnancy among Canadian women.

## Trial status

This trial is currently open for enrollment, with the first enrollments having commenced on 5 October 2010. Enrollment in the non-intervention group (those women not choosing to use an IUD) has been slower than expected but full enrollment is anticipated in late 2012.

## Abbreviations

BC0: British Columbia a province in Canada; CSQ: Contraception satisfaction questionnaires; FlexiT380 and Flexi T: Copper Intrauterine Device (“Flexi-T380(+)”® Prosan, The Netherlands); IUD: Intrauterine device; NovaT200 and NovaT: Copper Intrauterine Device (“Nova-T200”® Bayer Inc, Canada); PCR: Polymerase chain reaction; RCT: Randomized controlled trial; UBC: University of British Columbia; WHRI: Women’s health research institute.

## Competing interests

The authors declare that they have no competing interests.

## Authors’ contributions

WN, JK, RB, CT, and JC made substantial contributions to conception and design of this study. WN and AA will contribute to acquisition of data, and all authors will contribute to the analysis and interpretation of data. WN, JK, RB, CT, JC and AA drafted the protocol and protocol manuscript, and all authors contributed to critical revision for important intellectual content. All authors have read and approved the final manuscript.

## Authors’ information

All authors work within the Contraception and Abortion Research Team of the University of British Columbia (UBC). This team is supported by the Women’s Health Research Institute (WHRI) of British Columbia Women’s Hospital and Health Centre, Vancouver, Canada. WVN was supported by the Clinician Scholar's Program within the UBC Faculty of Medicine at the time of study inception, and is currently supported as a “Strategic Training Fellow in Interdisciplinary Primary Health Care Research” by the Canadian Institutes of Health Research Strategic Training Program – Transdisciplinary Understanding and Training on Research – Primary Health Care.
